# The impact of varying doses of moringa leaf methanolic extract supplementation in the cryopreservation media on sperm quality, oxidants, and antioxidant capacity of frozen-thawed ram sperm

**DOI:** 10.1007/s11250-022-03344-y

**Published:** 2022-10-13

**Authors:** Islam E. El-Seadawy, Mohamed S. Kotp, Amal M. Abo El-Maaty, Aya M. Fadl, Hossam R. El-Sherbiny, Elshymaa A. Abdelnaby

**Affiliations:** 1 Animal Reproduction and Artificial Insemination Department, Veterinary Research Institute, National Research Centre, Giza, Egypt; 2 Theriogenology Department, Faculty of Veterinary Medicine, Cairo University, Giza, 12211 Egypt

**Keywords:** MLME, Rams, Post-thaw semen quality, Oxidants–antioxidants, Trace minerals

## Abstract

To increase rams’ post-thaw semen quality following cryopreservation, this study used enriched Tris-based diluent with varying amounts of moringa leaf methanolic extract (MLME). The antioxidant activity, total phenolic, and total flavonoid content were all assessed in MLME. The sperm of five healthy Awassi rams were collected, divided into 4 equal aliquots, and diluted [1:5; (v/v)] in Tris-citrate-glucose extender supplemented with 0.48, 0.56, and 0.64 mg MLME/ml or without MLME supplementation (control). The percentages of sperm total motility (STM, %), sperm progressive motility (SPM, %) and viability (V, %), abnormal morphology (AM, %), membrane functional integrity (MFI, %), and acrosome integrity (AI %) were measured. Malondialdehyde (MDA), nitric oxide (NO), ascorbic acid (AA), superoxide dismutase (SOD), glutathione peroxidase (GPx), total cholesterol (TC), low-density lipoproteins (LDL), lactate dehydrogenase (LDH), alkaline phosphatase (ALP), zinc (Zn), and copper (Cu) were measured. The total phenolic gallic acid and flavonoid catechin (equivalent) contents were 19.78 mg/g and 11.94 mg/g, respectively. 2,2-Diphenyl-1-picrylhydrazyl (34.37 mM TE/g) and 2,2′-azino-bis/3-ethylbenzothiazoline-6-sulfonic acid (53.47 mM TE/g) were found in MLME. MLME had a 64.59 mM TE/g ferric-reducing power. In comparison to control, the addition of 0.64 mg/ml MLME to Tris-based extender resulted in the highest (*P* < 0.001) STM (55.22 ± 0.98), SPM (45.41 ± .70), SV (60.01 ± 1.05), MFI (75.23 ± 0.77), and AI (73.13 ± 0.72) and the lowest (*P* < 0.001) AM (21.34 ± 0.72) values. In comparison to the control, the addition of 0.56 mg/ml semen extender resulted in lower STM, SPM, SV, MFI, and AI with higher AM percentages. MDA (*P* = 0.03), NO (*P* = 0.012), CHO (*P* = 0.0001), and LDL (*P* = 0.004) were reduced by 0.64 mg/ml MLME, while AA (*P* = 0.017) and SOD (*P* = 0.0001) were elevated. In conclusion, the highest copper (*P* = 0.006) and lowest zinc concentrations in MLME (0.48 mg/ml extender) deteriorated the post-thaw semen quality, prompting us to suggest the addition of 0.64 mg MLME to rams’ Tris-based semen extender.

## Introduction

Artificial insemination (AI) plays a substantial role in improving breeding efficiency and livestock productivity. Among the major determinants of AI program success, germ cell cryopreservation comes in the advanced position. Sperm cells, during the stages of freezing and thawing processes, face dramatic challenges to withstand the adverse effects of extender hypertonicity and ice crystallization (Aisen et al. 2002), as well as membranes (plasma, acrosomal, and nuclear) lipid peroxidation (Bucak et al. 2009). Although reactive oxygen species (ROS), in optimal levels, are integrated into the physiological capacitation reaction and fertilizing potential of spermatozoa, their higher levels generated during the cryopreservation process provoke oxidative stress (OS) with subsequent sperm membrane lipid peroxidation and protein denaturation cascades which, in turn, disrupt its fluidity, intactness, and fertilizing capacity (Sarıözkan et al. 2009). Ram spermatozoa contain, in its plasma membrane, a high level of polyunsaturated fatty acids (PUFA), which make them more vulnerable to lipid peroxidation than other species. Mitigation of the harmful impacts of OS during sperm cryopreservation has been a primary goal for AI specialists in the last few decades. Supplementation of the cryopreservation medium with antioxidants, either synthetic or natural, has been proved to be helpful for sperm viability and fertilizing potential (Tuncer et al. 2011)

Natural extracts and infusions from fruits and vegetables attracted researchers’ interest to supplement semen extenders for preserving animal sperm (El-Sheshtawy and El-Nattat 2020; El-Nattat et al. 2011). Plants and their products are possible sources of phytochemicals, which have antioxidant activity, and hence prevent free radicals’ generation (Khalafalla et al. 2010). Drumstick tree, horseradish tree, and *Moringa oleifera* Lam (Family: Moringaceae) are extremely prized plants in tropical and subtropical nations where they are usually grown (Bakre et al. 2013; Taher et al. 2017). The leaves contain higher concentrations of protein, carotene, vitamins (such as A, B, C, E, riboflavin, nicotinic acid, folic acid, and pyridoxine), amino acids, minerals, and numerous antioxidant compounds (polyphenols, flavonoids, proanthocyanidins, and flavonols) (Anwar et al. 2007; Khalafalla et al. 2010). These polyphenols, flavonoids, proanthocyanidins, flavonols, vitamin C and E, carotene, zinc, and selenium have all been reported to have high antioxidant properties (Dhalaria et al. 2020). Heat-stressed rats treated with *Moringa oleifera* leaves extract showed improvements in the perspectives of sexual activity, spermogram, reproductive hormones, and antioxidant capacity (Prabsattroo et al. 2015; Afolabi et al. 2013; Venkatesh et al. 2019). In rabbits, dietary supplementation of *Moringa oleifera* leaf extract (MOLE) (Ajuogu et al. 2019), orally (El-Desoky et al. 2017), or added to semen extenders (El-Seadawy et al. 2017) improved sperm motility, viability, and seminal antioxidant capacity. In another study, MOLE supplemented orally to Barki rams did not improve spermatozoa motility and viability over 64 days of the administration, but it improved their antioxidant status (Shokry et al. 2020). Higher post-thaw sperm motility and viability were reported following fortification of Barki ram freezing extender with MOLE (Shokry et al. 2021). In rams, *Moringa oleifera* seed methanolic extract improved post-thaw semen motility and antioxidant capacity (Carrera-Chávez et al. 2020). The biochemical analysis of chilled and frozen ram semen was reported (Abd El-Hamid 2019). There was a lack of information about the defined composition and antioxidant potential of the MOLE that supported the positive effects on sperm viability. Besides, the reports described a full biochemical analysis of the post-thaw sperm environment are scarce. Therefore, this study aimed, for the first time, to provide a full biochemical analysis of the freezing outcomes including oxidants, antioxidants, enzymes, and minerals profiles, as well as a defined detailed MOLE composition, and moreover, to specify the most appropriate dose of MOLE enrichment of the Tris-based diluent to optimize high post-thaw semen quality.

## Material and methods

### Samples and chemicals

#### Moringa oleifera leaves samples

*Moringa oleifera* leaves were purchased from the Moringa Production Unit (National Research Centre) and then air-dried, preserved in the shade for 7 days, pulverized into powder, passed through a mesh sieve-40, and stored in plastic bags until needed.

#### Phenolic acid standards

Gallic, protocatechuic, gentisic, chlorogenic, vanillic, caffeic, syringic, p-coumaric, ferulic, sinapic, rosmarinic, and cinnamic acid, catechin, and apigenin were collected From Sigma–Aldrich, Inc. (Louis, USA).

#### Radical precursor

Sigma–Aldrich, Inc., supplied DPPH (2,2-diphenyl-1-picrylhydrazyl), ABTS (2,2-azino-bis/3-ethil-benothiazoline-6-sulfonic acid), TPTZ (2,4,6-tripyridyl-s-triazine), and Folin–Ciocalteu reagent (St. Louis, USA).

#### Solvents and other chemicals

Aldrich Chemical provided acetonitrile (HPLC grade) (GmbH & Co KG, Steinheim, Germany). Tedia Company, 45014, USA, provided petroleum ether, diethyl ether, ethyl acetate, tetrahydrofuran, and methanol (analytical grade). Sodium hydroxide, potassium persulfate, dinitrosalicylic acid, aluminum chloride, sodium carbonate, hydrochloric, sulfuric, and acetic acids were included in this experiment.

### Preparation of the MLME

The raw materials (30 g) were homogenized with 300 ml methanol (1:10 w/v) and agitated at room temperature to make moringa leaf methanolic extract (MLME). For 24 h, the solution was maintained at 4°C. The resulting extract was filtered and then dried under decreased pressure at 40°C using a rotary evaporator, and the final yield of extract was reported (Roopalatha and Nair 2013). The extract was reconstituted in 10 ml DMSO and stored at −80°C until further use.

### Major phytochemicals in the prepared extract

#### Evaluation of total phenolic content

The total phenolic content was determined according to the Folin–Ciocalteu procedure (Zilic et al. 2012). Total phenolic content was calculated as mg/g gallic acid equivalent using equation *Y* = 0.034 *x* + 0.111, *R*^2^ = 0.999, where *X* is the absorbance and *Y* is the gallic acid equivalent (mg/g).

#### Total flavonoid content determination

The total flavonoid content was determined according to Zilic et al. (2012) using aluminum chloride assay. Total flavonoid contents were performed as equivalent (mg/g) using the following *Y*= 0.012 *x* + 0.008, *R*^2^ = 0.998, where *x* is the absorbance and *Y* is the catechin equivalent (mg/g).

### Antioxidant activity determination of prepared extracts

#### DPPH radical scavenging activity

The stable DPPH was used to measure the free radical scavenging ability of extracts, according to Hwang and Do Thi (2014), with a Trolox curve (*Y*= 0.4841 *x* + 2.113, *R*^2^ = 0.999, where *x* is the inhibition percent and *Y* is the Trolox equivalent mg/g). The results are given in milligrams of Trolox equivalent per gram of extract.

#### ABTS radical scavenging activity

ABTS radical scavenging capacity of extract was determined according to Hwang and Do Thi (2014) with a curve using *Y*= 2.965 *x* + 0.693, *R*^2^ = 0.999, where *x* is the inhibition percentage and *Y* is the Trolox equivalent mg/g). Results are expressed as mg of Trolox equivalent per g of extract.

### Ferric-reducing activity power (FRAP) assay

The FRAP test was performed using *Y*= 0.041 *x* + 0.006, *R*^2^ = 0.999, where *x* is the inhibition percentage and *Y* is the Trolox equivalent mg/g), as described by Hwang and Do Thi (2014). The results are given in milligrams of Trolox equivalent per gram of extract.

### Separation and identification of phenolic acids by high-performance liquid chromatography (HPLC)

Samples were automatically fed into a diode array detector-equipped HP 1100 series HPLC system (Hewlett-Packard, GmbH, Germany) (DAD). The primary peaks’ absorption spectra were measured at 280 and 320 nm. A Xterra RP18 reverse phase column (4.6 mm250 mm) with a spherical particle size of 5 m was used in the HPLC system, which was maintained at 25°C. The mobile phase contains 1% formic acid (A) and acetonitrile (B), and the elution gradient was 2 to 100% (B) in 40 min at a flow rate of 0. ml/min at a temperature of 25°C. Gayosso-García Sancho et al. (2011) used a 20-l injection volume.

### Animal management and semen collection

Five fertile and clinically healthy Awassi rams, 42.35±3.25 months of age and 55.25±2.35 kg body weight, exposed to natural daylight, ambient temperature, and relative humidity of the breeding season of rams in Egypt (winter season), were used in the current work. They received their maintenance requirement of feed following NRC (1985). The ration (1.25 kg/day) was introduced to each ram, composed of 850 g of green fodder and wheat straw with 400 g-formed concentrates (Abdelnaby 2020).

Semen samples were collected using an artificial vagina (4°C) of the rams twice weekly for 2 months. Immediately after collection, the semen samples were transferred to the laboratory at 37°C for examination and processing. For exclusion of the rams’ variations, the obtained ejaculates were pooled after assessment of the mass motility. Semen samples had mass motility scores (1–5) over 4 were pooled. Semen quality parameters including sperm progressive motility (%) and sperm cell concentration (10^9^ cell/ml; modified Neubauer hemocytometer) were examined in the pooled samples for dilution rate calculation. The inclusion criteria for fitting the samples for further freezing procedures were (1) progressive motility ≥ 75% and (2) sperm cell concentration ≥ 2.7 × 10^9^ sperm cell/ml.

Pooled samples were allotted into 4 equal parts; each part was diluted 1:5 (v/v) with a Tris-citrate-glucose extender. The used freezing extender was composed of Tris (hydroxyl methyl amino methane; 3.634 g), glucose (0.5 g), citric acid (1.99 g), egg yolk (15 ml), glycerol (7 ml), streptomycin sulfate (100 mg), and penicillin G sodium (100.000 IU) with the addition of glass bi-distilled water till 100 ml as a final volume (Evans and Maxwell 1987). MLME at concentrations of 0.48 mg, 0.56 mg, and 0.64 mg was added to each ml of the diluent (El-Seadawy et al. 2017) or without MLME supplementation (control). Both supplemented and control diluted spermatozoa were cooled for 2 h at 5°C, equilibrated for 15 min at 5°C, manually packed in 0.25 ml plastic French straws, and sealed ultrasonically (cryosealer, Minitube, Germany). After that, the straws were evenly distributed on a tray at 6.5 cm above the liquid nitrogen for 10 min and then immersed deeply into liquid nitrogen for long storage. Frozen straws were thawed by emerging two straws representing each treatment in a water bath adjusted at 40°C for 30 s (Salamon and Maxwell 2000).

### Evaluation of post-thaw sperm total and progressive motility

Thawed spermatozoa were subjectively assessed immediately after thawing for total motility (magnification: 100×) and progressive motility (magnification: 400×) and expressed in percent (0–100) by using a heated stage (37 °C) optical microscope (Olympus BH-2, Olympos Optical Co. Ltd., Japan). The rectilinear forward motility was considered only, whereas other types of local motilities were excluded. The sperm motility assessment was based on 5 different microscopic fields/slides in duplicate smears for validity assurance.

### Sperm viability and total abnormalities assessment

Alive sperm percentage was examined by using the supra-vital eosin-nigrosin staining method (Evans and Maxwell 1987). Each sample was stained by mixing a 4 µl of frozen-thawed semen with 20 µl of the stain (1.67 g eosin, 5 g nigrosin, and 2.9 g sodium citrate dihydrate/100 ml distilled water) on a pre-warmed (37 °C) glass slide. In at least 5 different microscopic fields, a sum of 200 spermatozoa was examined randomly, for the sperm’s ability to stain refusal, using a bright-field microscope (magnification: 1000× oil immersion). Structurally intact sperm appeared whitish (not stained), whereas deteriorated sperm (those with damaged plasma membrane) stained and appeared red. Sperm cell abnormalities including head defects, cytoplasmic droplets, coiled tails, and mid-piece deformities were evaluated in the same slide used for sperm viability evaluation in at least 5 different microscopic fields (magnification: 1000× oil immersion). The total sperm abnormalities were expressed as a percentage (0–100). The examinations were routinely performed in duplicate smears for more precise assessment.

### Membrane functional integrity

Hypo-osmotic swelling test (HOST) was used for sperm osmotic resistance assessment; intact viable sperm showed more resilience toward the hypo-osmotic solution and appeared swollen with curled tail (Revell and Mrode 1994). Briefly, a tiny drop (40 μl) of thawed semen sample was mixed with 400 μl of hypo-osmotic solution (0.9-g fructose plus 0.49 g sodium citrate per 100 ml of distilled water, 100 mOsm/kg). The mixture was incubated at 37°C for 45 min. A tiny drop of the mixture (5 µl) was hanging on a microscope slide, coverslipped, and was immediately examined by a bright-field microscope (400× magnification). Spermatozoa with functional plasma membranes appeared swollen with coiled tails, while inactive spermatozoa remained unchanged. Sums of 200 spermatozoa in duplicate smears were examined in five different microscopic fields. The number of sperm that appeared swollen with coiled tails was recorded.

### Acrosome integrity

The intactness of the sperm acrosome was examined using a patent-specific stain (Spermac) according to a previous study (Ghallab et al. 2019). In brief, air-dried sperm smears were fixed in formalin (10%) for 10 min. The staining procedures were performed via passage of the fixed slides into the stain solutions (A, B, and C) for 1 min each at room temperature and then let to air dry. The air-dried slides were assessed using an oil immersion lens (1000× magnification). As routinely performed, duplicate smears were prepared, and a total of 200 spermatozoa were examined, and the sperm cells with intact acrosomes were calculated and expressed in percent (0–100).

### Biochemical evaluation

After thawing the straws and separating the supernatant, commercial kits were used to measure lipid peroxide product (malondialdehyde (MDA) nmol/ml) (Fathi et al. 2021), nitric oxide (NO) (umol/l), glutathione peroxidase (GPx) (mU/ml), superoxide dismutase (SOD) (U/ml), ascorbic acid (AA) (mg/l), zinc (Zn) (μg/dl), copper (Cu) (μg/dl), and total cholesterol (TC) (mg/dl) (Biodiagnostics, Tahreer St., Dokki, Giza, Egypt). Lactate dehydrogenase (LDH) (U/l) and alkaline phosphatase (ALP) (IU/l) (MG, Science, and Technology Center “STC”) were tested colorimetrically utilizing a spectrophotometer, with a sensitivity of 1U/100 ml and a 0.56% and 1.1% intra- and inter-test precision, respectively

### Statistical analysis

The obtained data were expressed as mean ± SEM. The effects of different concentrations of the MLME added to the semen diluent on the semen parameters were tested using a one-way analysis of variance (ANOVA). Duncan’s multiple range test was used to differentiate between significant means at *P*<0.05. Pearson’s correlation coefficient was also processed. All the statistical analyses were performed using IBM-SPSS/PC version 20.0 (2016).

## Results

**Fig. 1:**
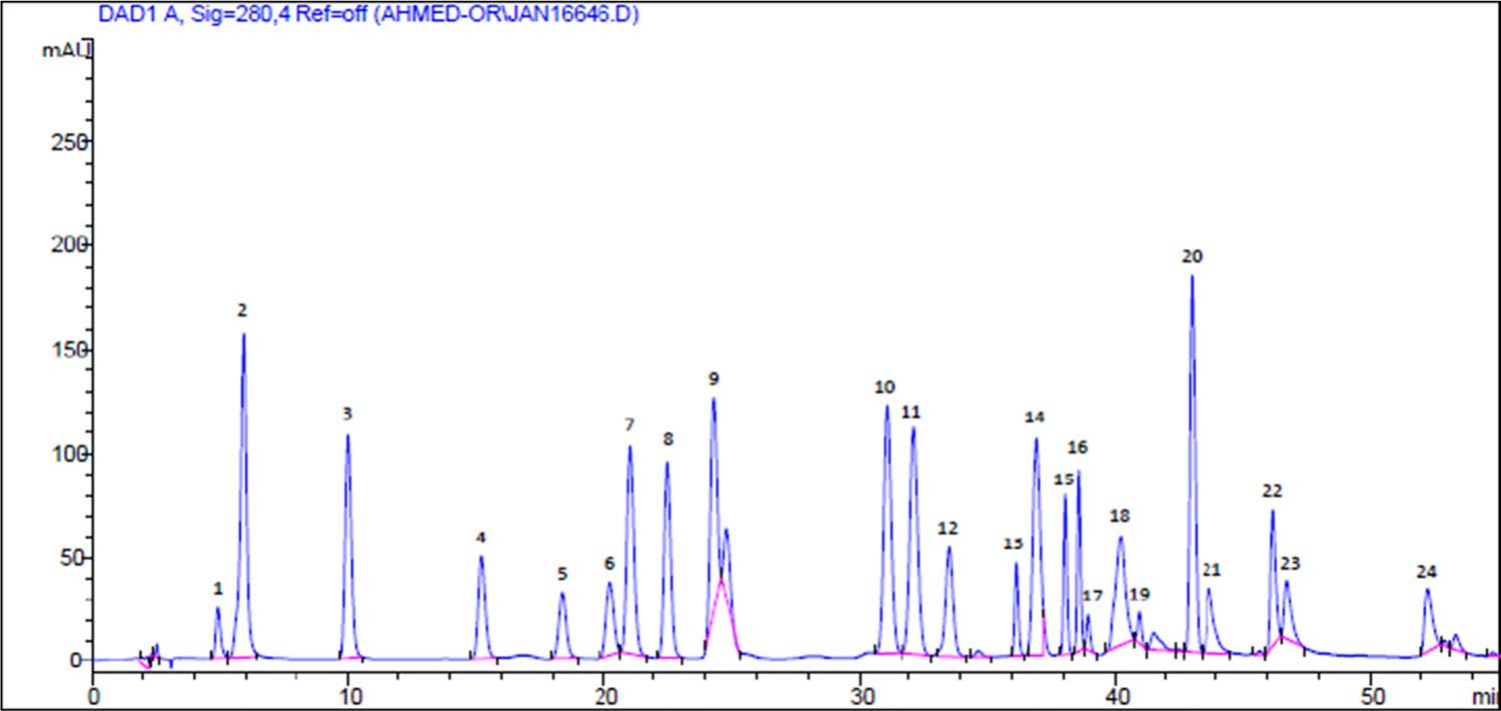
HPLC chromatograms of standard metabolites showing signal from diode array detector at a wavelength of 280 nm. Peak 1, pyrogallol; 2, gallic acid; 3, protocatechuic acid; 4, *P*-hydroxybenzoic acid; 5, catechin; 6, chlorogenic acid; 7, caffeic acid; 8, syringic acid; 9, vanillic acid; 10, scopolamine; 11, ferulic acid; 12, sinapic acid; 13, rutin; 14, p-coumaric acid; 15, naringenin; 16, hesperidin; 17, apeginin-7-glucoside; 18, myricetin; 19, rosmarinic acid; 20, cinnamic acid; 21, quercetin; 22, apigenin; 23, kaempferol; 24, chrysin

MLME has a total phenolic content of 19.78 mg GAE/g extract. The total flavonoid concentration of the extract is 11.94 mg CE/g. MOLE antiradical activity against ABTS is 53.47 mM TE/g, while MOLE antiradical activity against DPPH is 34.37 mM TE/g. Using FRAP method, the reducing power was determined to be 64.59 mM TE/g/g. HPLC analysis was used to compare the results to 24 standardized metabolites (Table 1, Fig. 1).

**Table 1:** HPLC analysis of polyphenolic compound MLME

Compound	Retention time (min)	Concentration (µg/g extract)
Pyrogallol	4.90	1104.16
Rutin	36.18	8278.57
*P*-Hydroxybenzoic acid	15.22	671.18
Naringenin	38.07	186.80
Chlorogenic acid	20.28	36.06
Caffeic acid	21.08	218.44
Hesperidin	38.60	544.57
Cinnamic acid	41.52	32.95
Vanillic acid	24.82	39.28
Apeginin-7-glucoside	38.96	214.01
Myricetin	40.24	190.40
Kaempferol	46.22	37.31
Rosmarinic acid	40.95	1364.81

**Fig. 2:**
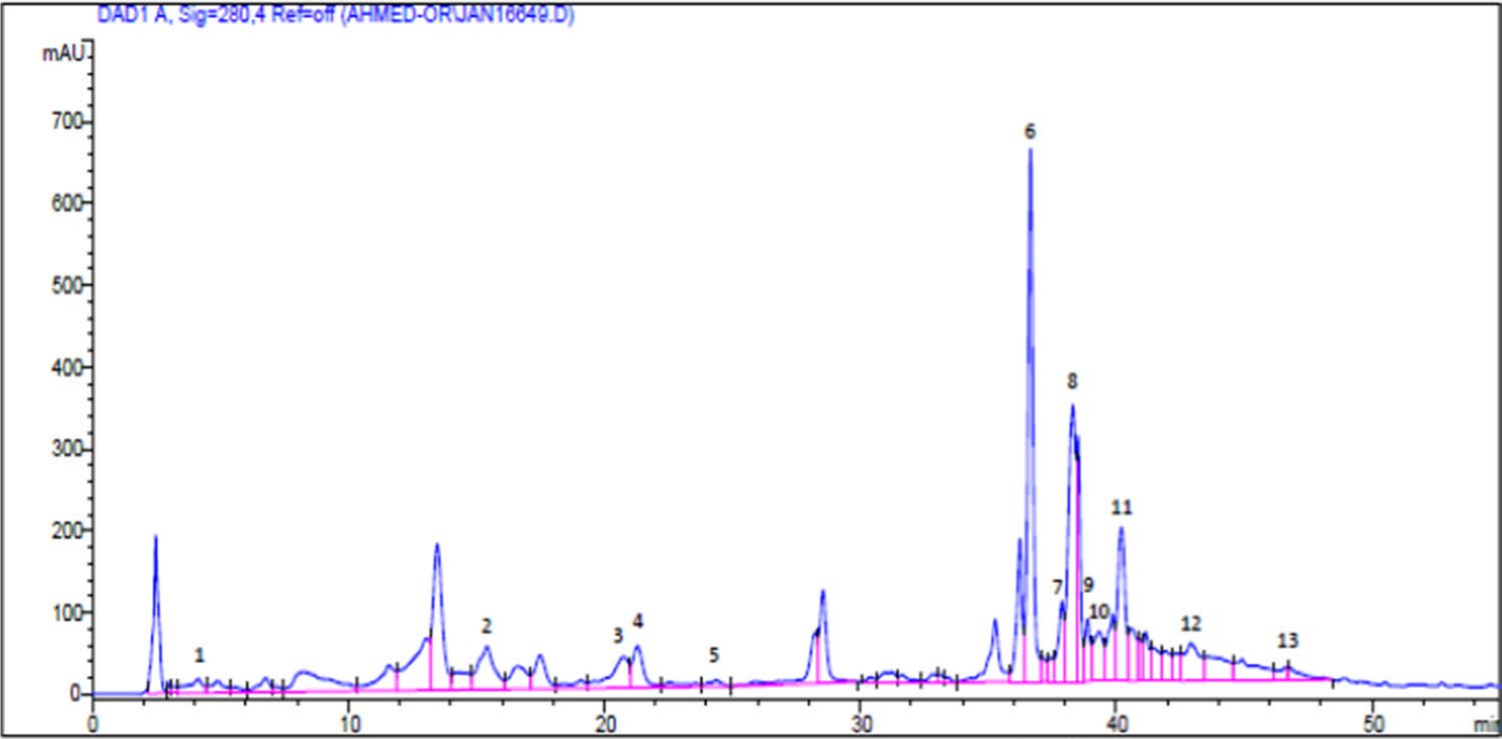
HPLC chromatograms of moringa leaf extract showing signal from diode array detector at a wavelength of 280 nm. Peak 1, pyrogallol; 2, *P*-hydroxybenzoic acid; 3, chlorogenic acid; 4, caffeic acid; 5, vanillic acid; 6, rutin; 7, naringenin; 8, hesperidin; 9, apeginin-7-glucoside; 10, myricetin; 11, rosmarinic acid; 12, cinnamic acid; 13, kaempferol

Rutin (8.278 mg), rosmarinic acid (1.364 mg), and pyrogallol are the most effective chemicals in each gram of the MLME extract (1.104 mg). P-Hydroxybenzoic acid (0.671 mg), hesperidin (0.545 mg), caffeic acid (0.218 mg), apeginin-7-glucoside (0.214 mg), myricetin (0.190 mg), and naringenin (0.190 mg) are some of the other substances with smaller levels (0.187 mg). Vanillic acid (0.039 mg), kaempferol (0.037 mg), chlorogenic acid (0.036 mg), and cinnamic acid (0.033 mg) were all detected in trace amounts (Fig. 2).

The data describing the effect of MLME on the post-thaw sperm quality parameters are presented in Table 2. Marked increases (*P* = 0.0001) were noted in the post-thaw MLME supplemented spermatozoa (0.56 and 64 mg/ml) in the perspective of STM (45.23 0.72 and 55.22± 0.98, respectively), SPM (35.32± 0.99 and 45.41± 0.70, respectively), V (49.22± 0.71 and 60.01± 1.05, respectively), MFI (70.24± 0.88 and 75.23± 0.77, respectively), and AI (66.34± 0.83 and 73.13 ±0.72, respectively) compared to 0.48 mg/ml group (35.10± 0.81, 25.08± 0.81, 38.44± 0.69, 61.22± 0.76, and 62.03± 0.81, respectively) and control group (40.03 ± 0.68, 30.33± 0.62, 40.645± 0.76, 65.32± 0.79, and 62.03^b^± 0.81, respectively). Post-thaw AM was significantly decreased in MLME-treated spermatozoa especially at concentrations of 0.56 and 0.64 mg/ml (26.34± 0.69 and 21.34± 0.72, respectively) in relation to 0.48 mg/ml and control groups (35.37± 0.46 and 30.42± 0.89, respectively). Lipid peroxide products were reduced (*P*=0.03) in all diluents enhanced with MLME (malondialdehyde (MDA)). When 0.48mg/ml was added to semen diluents (Table 3), the lowest MDA (4.31±0.17 μmol/ml) was detected compared to the control (6.67±1.03 μmol/ml). When 0.64 mg MLME was added (Table 3), the lowest nitric oxide (NO) values (*P*=0.012) were observed, although the other two concentrations did not differ from the control. The activity of superoxide dismutase (SOD) increased (*P*=0.0001) in all three MLME concentrations, with the highest activity shown when 0.56mg MLME (7125±423 U/ml) was used and the lowest activity seen when 0.64mg MLME (4250±192 U/ml) was used. In all semen diluents supplemented with MLME concentrations, zinc concentrations increased thrice (*P*=0.0001) (Table 3).

**Table 2:** Sperm total motility percentages (STM %), sperm progressive motility percentages (SPM %), sperm viability percentages (V %), abnormal morphology (AM %), membrane functional integrity percentages (MFI %), and acrosome integrity percentages (AI %) of frozen-thawed ram semen supplemented with *Moringa*. Data are presented as mean ± standard error of mean (SEM)

Frozen-thawed semen characteristics	0.0 mg/ml	0.48 mg/ml	0.56 mg/ml	0.64 mg/ml	*P* value
Sperm total motility (STM %)	40.03^b^ ± 0.68	35.10^a^ ± 0.81	45.23^c^ ± 0.72	55.22^d^ ± 0.98	0.0001
Sperm progressive motility (SPM %)	30.33^b^ ± 0.62	25.08^a^ ± 0.81	35.32^c^ ± 0.99	45.41^d^ ± 0.70	0.0001
Viability (V %)	43.25^b^ ± 0.76	38.44^a^ ± 0.69	49.22^c^ ± 0.71	60.01^d^ ± 1.05	0.0001
Abnormal morphology (AM%)	30.42^c^ ± 0.89	35.37^d^ ± 0.46	26.34^b^ ± 0.69	21.34^a^ ± 0.72	0.0001
Membrane functional integrity (MFI %)	65.32^b^ ± 0.79	61.22^a^ ± 0.76	70.24^c^ ± 0.88	75.23^d^ ± 0.77	0.0001
Acrosome integrity (AI %)	62.03^b^ ± 0.81	57.42^a^ ± 0.91	66.34^c^ ± 0.83	73.13^d^ ± 0.72	0.0001

Means with different superscripts (a, b, c, d) in the same row are significantly different at *P* < 0.05

*n* = 16

**Table 3:** Seminal plasma lipid peroxide products as malondialdehyde (MDA), nitric oxide (NO), ascorbic acid (AA), glutathione peroxidase (GPx), superoxide dismutase (SOD), zinc(Zn), copper (CU), total cholesterol (TC), low-density lipoproteins (LDL), lactate dehydrogenase (LDH), and alkaline phosphatase (ALP). Data are presented as mean ± standard error of mean (SEM)

Variables	0.0 mg/ml	0.48 mg/ml	0.56 mg/ml	0.64 mg/ml	*P* value
MDA μmol/ml	6.67^b^ ± 1.03	4.31^a^ ± 0.17	5.01^a^ ± 0.30	4.96^a^ ± 0.21	0.03
NO μmol/L	50.00^b^ ± 2.78	47.36^b^ ± .39	48.29^b^ ± 0.55	43.01^a^ ± 0.65	0.012
AA g/L	824.37^a^ ± 14.97	891.61^b^ ± 11.39	846.79^a^ ± 21.45	869.06^ab^ ± 8.81	0.017
GPx mU/ml	82.14^ab^ ± 11.21	47.55^a^ ± 2.44	97.27^bc^ ± 13.07	138.34^c^ ± 27.15	0.003
SOD U/ml	2625^a^ ± 244	4750^b^ ± 297	7125^c^ ± 423	4250^b^ ± 192	0.0001
Zn μg/dl	94.41^a^ ± 0.31	277.85^b^ ± 0.98	285.71^b^ ± 1.70	296.48^c^ ± 5.21	0.0001
Cu μg/dl	540.25^a^ ± 52.5	934.95^b^ ± 146.01	440.47^a^ ± 72.7	605.79^a^ ± 99.88	0.006
TC mg/dl	160.67^b^ ± 10.02	117.87^a^ ± 5.06	127.07^a^ ± 2.91	113.20^a^ ± 2.14	0.0001
LDL mg/dl	117.29^b^ ± 9.35	94.99^a^ ± 6.33	93.35^a^ ± 4.25	84.56^a^ ± 2.44	0.004
LDH U/L	4857^a^ ± 104	5686^b^ ± 81	6990^c^ ± 443	5918^b^ ± 75	0.0001
ALP IU/L	137.98^ab^ ± 0.79	136.06^a^ ± 0.57	137.76^ab^ ± 4.01	149.75^b^ ± 7.78	NS

Means with different superscripts (a, b, c, d) are significantly different at *P* < 0.05, non-significant (NS) (*n* = 16)

The addition of 0.48 mg MLME to semen diluents contained the highest (*P*=0.006) copper concentrations (934.95±146.01 μg/dl). Total cholesterol (TC) (*P*=0.0001) and LDL (*P*=0.004) had decreased in all MLME concentrations added to semen extenders. LDH increased (*P*=0.0001) in all semen diluents supplemented with MLME with the maximum increase (6990±192 U/ml) was noticed when 0.56 mg MLME was added (Table 3). Alkaline phosphatase activity did not vary after adding the three moringa concentrations (Table 3). Zinc (Zn) (μg/dl) tended to correlate positively with STM % (*r*=0.27; *P*=0.066) and negatively with AM % (r= −0.32; *P*<0.08), correlated with V % (*r*=0.37; *P*=0.045), MFI % (*r*=0.39; *P*<0.034) (Table 4). SOD correlated negatively with STM % (*r*=−0.37; *I*=0.047), SPM % (*r*=−0.43; *P*=0.019), and MFI % (*r*=−0.42; *P*=0.022), and AI % (*r*=−0.43; *P*=0.016) tended to correlate with V% (*r*=−0.34; *P*=0.063). ALP correlated with STM % (*r*=0.57; *P*=0.001), SPM % (*r*=0.48; *P*=0.007), MFI % (*r*=0.41; *P*=0.023), and AI MFI (*r*=0.42; *P*=0.02) but correlated negatively with V% (*r*=−0.56; *P*=0.001) and AM % (*r*=−0.49; *P*=0.006).

**Table 4:** Pearson correlation coefficient between post-thaw semen characteristics with copper (Cu), total cholesterol (TC), low-density lipoproteins (LDL), nitric oxide (NO), alkaline phosphatase (ALP), ascorbic acid (AA), superoxide dismutase (SOD), glutathione peroxidase (GPx), and lactate dehydrogenase (LDH)

Semen parameters	MDA	Zinc	Cu	ALP	SOD
Total motility %	0.21	0.34^#^	− 0.24	0.57^**^	− 0.37*
Progressive motility%	0.20	0.27	− 0.22	0.48^**^	− 0.43*
Live sperm%	0.18	0.37^*^	− 0.26	− 0.56^**^	− 0.34^#^
Abnormal morphology %	− 0.18	− 0.32^#^	0.21	− 0.49^**^	0.29
HOST	0.14	0.39^*^	− 0.27	0.41^*^	− 0.42*
Acrosome	0.20	0.31	− 0.26	0.42^*^	− 0.43*

^**^ Significant at *P* < 0.01, * significant at *P* < 0.05, # tended to be significant at *P* > 0.05

## Discussion

Semen extenders must be added with antioxidants to combat the reactive oxygen species (ROS) that is created during semen cryopreservation processing to acquire the excessive fertilizing capacity of post-thaw semen (Capucho et al. 2012; Rosato and Iaffaldano 2011; Fadl et al. 2022a). ROS depletes ATP, resulting in inadequate axoneme phosphorylation and lipid peroxidation, both of which reduce motility and survival (Bansal and Bilaspuri 2010; El-Sherbiny et al. 2022a). Antioxidants, vitamins, amino acids, and minerals are all located in moringa leaves (Khalafalla et al. 2010). *Moringa* carries antioxidants consisting of phenols, flavonoids, proanthocyanidins, flavonols, diet C, diet E, carotene, zinc, and selenium (Vergara-Jimenez et al. 2019). The phenolic content material of MLME on this examination is like others (Sreelatha and Padma 2009; Atawodi et al. 2010). The methanolic extract of our *M. oleifera* leaves decreased DPPH radicals much less than that of Sreelatha and Padma (2009). This decline is probably attributed to the polarity of the solvents and the plant’s geographic location (Sreelatha and Padma 2009). The typical polyphenolic concentration of MLME correlates with its antioxidant activity (Vieira et al. 2009) as antioxidant power is concentration-dependent (Siddhuraju and Becker 2003). In addition, the phenolic compounds in MLME are strong electron donors, and they may be able to stop the radical chain reaction by converting free radicals to stable products (Lobo et al. 2010). The present results showed that the addition of 0.56 and 0.64 mg MLME/ml Tris extender had appreciably maintained the rams’ post-thaw semen quality (motility, viability, membrane, and acrosome integrity, and diminished sperm abnormality). In agreement with our results, feeding 5% of freshly air-dried MOLE for 84 days improved the motility and viability of rabbit semen, SOD, and reduced MDA, but did not affect GPX (Jimoh et al. 2021). Similarly, male rabbits supplemented orally with 1.5 ml of water containing 50, 100, and 150 mg/kg BW ethanolic extract of MOLE confirmed the marked progress in sperm motility, viability, membrane, and acrosome integrities (El-Desoky et al. 2017). Rabbits supplemented orally 1.5-ml water containing 50, 100, and 150 mg/Kg BW showed an improvement in the sperm motility, viability, membrane, and acrosome integrities that was associated with low abnormal morphology % (El-Desoky et al. 2017). Moreover, when adding MLME from 0.4 to 4.0 mg/5ml rabbit semen extender that chilled for 72 h, it was found that the best concentration that maintained the highest sperm motility from 2 to 72 h was 2.0 mg (El-Seadawy et al. 2017). The gradual improvement in post-thaw sperm motility and viability observed in the current study with increasing MLME concentrations from 0.48, 0.56, to 0.64 was reversed when 0.5, 5.0, and 10.0 moringa seed methanolic extracts were added to ram semen (Carrera-Chávez et al. 2020) indicating that the improvement in post-thaw semen is concentration-dependent as >0.56 and >0.65 mg are more preferable and higher concentrations that were associated with lowered post-thaw antioxidant capacity and sperm membrane damage (Carrera-Chávez et al. 2020). However, in Barki rams, the increasing moringa leaf extract from 60 to 600 µg in the semen extenders could increase post-thaw sperm motility and viability but from 900 and 1200 µg/ml could reduce them to values lower than control, indicating that this improvement is dose dependent (Shokry et al. 2021). In contrary, the oral supplementation of Barki rams MOLE for 64 days did not alter sperm motility, viability, abnormal sperm, membrane functional integrity, and DNA fragmentation (Shokry et al. 2020). The addition of 100 and 500 µg/ml water extract of moringa leaves to soya-lecithin semen extender at 5 °C for 48 h declined sperm motility and viability with no change in the abnormal sperm, but the addition of 1000 µg/ml resulted in the same results of control (El-Harairy et al. 2016) indicating that the components of semen extender interact with the components of moringa extract and produce toxic compounds to sperm. In cattle, the post-thaw sperm motility and viability of semen extenders supplemented with 10 % and 2 0MOLE were improved, but higher concentrations from 30 to 50% did not, whereas the addition of 20–40% moringa increased the abnormal sperm and did not improve the functional membrane integrity (El-Sheshtawy and El-Nattat 2020) indicating that certain concentrations of moringa could improve post-thaw sperm motility and viability.

A significant lower in MDA and increase in SOD activity were discovered in the three concentrations of MLME of the current study which is like the addition of ten concentrations to chilled rabbit semen for 72 h (El-Seadawy et al. 2017). The testicular SOD increased and MDA when cryptorchid and non-cryptorchid rats were treated with ethanolic extract of MOLE (Afolabi et al. 2013). The significant decrease in the MDA and the increased SOD levels observed herein were noticed also when 300 and 600 µg/MOLE were added to Barki rams’ semen (Shokry et al. 2021). In contrast, to control non-treated rams, oral supplementation of rams with MOEE for 64 days raised SOD and decreased MDA in fresh seminal plasma and post-thaw semen (Shokry et al. 2020). In line with our findings, feeding male rabbits 5 % MOLE for 84 days decreased MDA while increasing SOD (Jimoh et al. 2021). NO is an oxygen-free radical molecule (Daghash et al. 2022) that shares in the regulation of spermatogenesis, sperm motility, and maturation (Abdelnaby et al. 2021; Abdelnaby et al. 2022; El-Sherbiny et al. 2022b). In agreement with our results, a low NO level plays an essential role in sperm motility (Du Plessis et al. 2010), but an increased level of NO decreases sperm motility and viability (Badade et al. 2011). The low levels of NO could participate in many physiological functions, but the higher ones could lead to much pathological damage to cells (Gholinezhad et al. 2020). So, NO oxidative actions may be regulated here through increasing superoxide with increasing MLME concentration that maintained high sperm motility and viability.

Supporting our findings, copper in high levels is toxic to spermatozoa and reduces both motility and viability (Knazicka et al. 2012). In other species, elevated copper concentrations reduced oxidative processes and glycolysis that in turn caused immobility and reduced viability (Pesch et al. 2006; Abdelnaby et al. 2022). The highest concentrations of zinc estimated in the semen extender supplemented with 0.64mg/ml MLME and their association with the highest semen quality with the lowest abnormal sperm percentage could be referred to its involvement in sperm maturation and correlation with an increased oxygen input and motility (Baldauf et al. 2003; Henkel et al. 2003; Fadl et al. 2022b, c). The concentration of MLME supplemented to rams’ semen in the current study showed different changes in the AA, GPx, and ALP, where the highest concentration indicated high AA, the highest GPx, and ALP, but the lowest one showed minimum GPx with the highest AA and did not change ALP. In contrast, to control non-treated rams, oral supplementation of rams with MOEE for 64 days increased seminal plasma AA, GPx, and ALP after the treatment but increased GPx, dropped ALP, and did not change AA in the post-thaw semen (Shokry et al. 2020). The significant increase of ascorbic acid in the post-thaw semen extenders supplemented with 0.48mg/ml with a slight insignificant increase when 0.56 and 0.64 mg/ml MLME was added in the current study was also noticed when 300 and 600 µg/ml of moringa leaf extract were added to Barki ram semen (Shokry et al. 2021). In contrast to our results where MLME did not increase the concentrations of ALP in the post-thaw semen extenders, it declined it when 300 and 600 µg/ml of moringa leaf extract were added to Barki ram semen (Shokry et al. 2021). The significant increase in GPx concentrations when 0.64mg MLME was included is in understanding with including 300 and 600 µg/ml of moringa leaf extricate to Barki rams’ semen extenders (Shokry et al. 2021).

The increased LDH in the three MLME concentrations added to Tris-based extenders and the comparison to the control one and the presence of very weak non-significant correlations with post-thaw semen parameters encountered in the current study agree with the non-significant increase of LDH by supplementing aqueous extract of moringa leaves to soya-lecithin-based semen extenders to Rahmani rams and chilling it for 48 h at 5°C (El-Harairy et al. 2016). This increase in LDH does not support the hypothesis that the metabolism of spermatozoa in an anaerobic condition depletes sperm energy and results in a significant negative correlation with pathomorphology of sperm as this may refer to the association of increased sperm metabolism with poor sperm characteristics depending on certain LDH isomer (Pesch et al. 2006). In sheep, LDH is predominantly localized in spermatozoa, and its leakage has been correlated with cell membrane damage. Though the increased LDH in the post-thaw semen showed no correlation with spermatozoa motility, viability, MFI, and AI, LDH leakage was observed at all stages of cryopreservation. LDH leakage therefore could be used as a marker of membrane damage both during pre-freeze and post-thaw incubations (Dhami and Kodagali 1990; Stéphenne et al. 2010). The increase of LDH in post-thaw semen may be attributed to the prevalent LDH leakage during cooling, freezing, and post-thaw incubation of spermatozoa that could be used as marker enzyme in the development of semen processing (Upreti et al. 1996,). The highest MLME concentration in the current study showed the lowest MDA, NO, high SOD, and GPx activity that was associated with the highest sperm characteristics and zinc which referred to the high content of rutin (quercetin-3-rhamnosyl glucoside) as it has a potent reducing power against the lipid peroxidation that exhibit strong DPPH, hydroxyl radical, and superoxide radical scavenging activity (Moretti et al. 2012; Moyo et al. 2012; Afolabi et al. 2013; Sadek 2014; Abarikwu et al. 2016).

## Conclusion

MLME obtained several antioxidant properties. Supplementing rams’ semen with MLME (0.64 mg/ml semen diluent) improved all post-thaw semen characteristics and biochemicals. The highest copper and lowest zinc levels in MLME (0.48 mg/ml extender) deteriorated the post-thaw semen quality, prompting us to suggest the addition of 0.64 mg MLME to rams’ Tris-based semen extender.

## Data Availability

Data sharing does not apply to this article as no new data were created in this study.
